# Comparative Proteomics of Salt-Tolerant and Salt-Sensitive Maize Inbred Lines to Reveal the Molecular Mechanism of Salt Tolerance

**DOI:** 10.3390/ijms20194725

**Published:** 2019-09-24

**Authors:** Fenqi Chen, Peng Fang, Yunling Peng, Wenjing Zeng, Xiaoqiang Zhao, Yongfu Ding, Zelong Zhuang, Qiaohong Gao, Bin Ren

**Affiliations:** 1 College of Agronomy, Gansu Agricultural University, Lanzhou 730070, China; 2 Gansu Provincial Key Lab of Aridland Crop Science, Lanzhou 730070, China

**Keywords:** maize, seedling root, salt stress, physiological response, proteomic analysis, iTRAQ, qRT-PCR

## Abstract

Salt stress is one of the key abiotic stresses that causes great loss of yield and serious decrease in quality in maize (*Zea mays* L.). Therefore, it is very important to reveal the molecular mechanism of salt tolerance in maize. To acknowledge the molecular mechanisms underlying maize salt tolerance, two maize inbred lines, including salt-tolerant 8723 and salt-sensitive P138, were used in this study. Comparative proteomics of seedling roots from two maize inbred lines under 180 mM salt stress for 10 days were performed by the isobaric tags for relative and absolute quantitation (iTRAQ) approach. A total of 1056 differentially expressed proteins (DEPs) were identified. In total, 626 DEPs were identified in line 8723 under salt stress, among them, 378 up-regulated and 248 down-regulated. There were 473 DEPs identified in P138, of which 212 were up-regulated and 261 were down-regulated. Venn diagram analysis showed that 17 DEPs were up-regulated and 12 DEPs were down-regulated in the two inbred lines. In addition, 8 DEPs were up-regulated in line 8723 but down-regulated in P138, 6 DEPs were down-regulated in line 8723 but up-regulated in P138. In salt-stressed 8723, the DEPs were primarily associated with phenylpropanoid biosynthesis, starch and sucrose metabolism, and the mitogen-activated protein kinase (MAPK) signaling pathway. Intriguingly, the DEPs were only associated with the nitrogen metabolism pathway in P138. Compared to P138, the root response to salt stress in 8723 could maintain stronger water retention capacity, osmotic regulation ability, synergistic effects of antioxidant enzymes, energy supply capacity, signal transduction, ammonia detoxification ability, lipid metabolism, and nucleic acid synthesis. Based on the proteome sequencing information, changes of 8 DEPs abundance were related to the corresponding mRNA levels by quantitative real-time PCR (qRT-PCR). Our results from this study may elucidate some details of salt tolerance mechanisms and salt tolerance breeding of maize.

## 1. Introduction

Salinity is one of the increasingly serious environmental and ecological problems, which threatens the limited soil resources on which human beings depend [1], and poses a major constraint on the sustainability of crop yields [2]. More than 830 million hectares of land are affected by salinity, accounting for over 6% of the world’s land area [3]. Therefore, how to maximize the use of this saline-alkaline land and reduce agricultural losses have become some of the most important tasks in the development of agricultural production. Salt stress can cause morphological, physiological, biochemical, and molecular changes in plants [4]. Many plant salt tolerance genes have been identified by transcriptome analysis, such as *OsNHX1* [5] and *OsHKT7* [6]. These data are useful for better understanding the mechanism of salt tolerance in plants, even though the expression level of mRNA does not directly correspond to protein abundance [7]. Proteomics can provide more direct information about cell metabolism, redox reactions, signal transduction and other responses to abiotic stress [8]. In recent years, the isobaric tags for relative and absolute quantitation (iTRAQ) has been used to study the proteomic characteristics and salt stress response proteins of plants [9,10].

Maize is one of the most important agricultural cash crops in the world because its raw materials are used in the production of human and animal food and biofuels [11,12,13]. Unfortunately, it is not a salt-tolerant crop [14]. Therefore, improving salt tolerance is especially important for increasing maize production. With the advent of the post-genomic era and the rapid development of proteomics technology, more and more scholars have applied proteomics to the study of the plant stress response. Kim et al. [15] studied the proteome of rice leaves under salt stress for 4 days and identified the proteins of the main metabolic processes such as photosynthetic carbon dioxide assimilation and photorespiration, which showed that there was a good correlation between salt stress-responsive proteins and leaf morphology. When Zhang et al. [16] studied the pathogen-reactive protein in cotton resistant variety CRI35 by iTRAQ-based proteomics methods, they found that cotton plants are active and multifaceted in defense against *Rhizoctonia solani* infection, by protein induction of various natural immune-related pathways. At present, physiology and proteomics using iTRAQ has been applied to the study of plant stress resistance, and a variety of salt-tolerant response mechanisms have been found, such as photosynthesis, signal transduction, ion balance, membrane transport, and reactive oxygen species scavenging systems [17,18]. The root is the main site of plant sensing of salt stress, and the sensitivity of the root to salt determines the production capacity of the whole plant [19]. Even so, there are few proteomics studies on the roots of maize response to salt stress.

Therefore, in this study, we performed a comprehensive comparative proteomic analysis of salt-sensitive P138 and salt-tolerant 8723 maize seedling roots under 180 mM salt stress using a high-throughput iTRAQ-based technique to explore salt-related proteins and molecular mechanisms.

Our study has found some proteins related to salt and revealed several important pathways related to salt reactions, such as lipid phosphate phosphatase 3, glutamate synthase 1, receptor-like protein kinase, cytochrome P450 11, Granule-bound starch synthase 1 and so on. The differences in salt tolerance between the two maize varieties were clarified at the protein level, and the molecular mechanisms involved in salt stress were fully understood. These results provide not only important information for our better understanding of the molecular mechanism of salt tolerance, but also basic information for the breeding of new varieties of salt tolerance in maize.

## 2. Results

### 2.1. Physiological Differences between P138 and 8723 under the Salt Treatments

**Figure 1:**
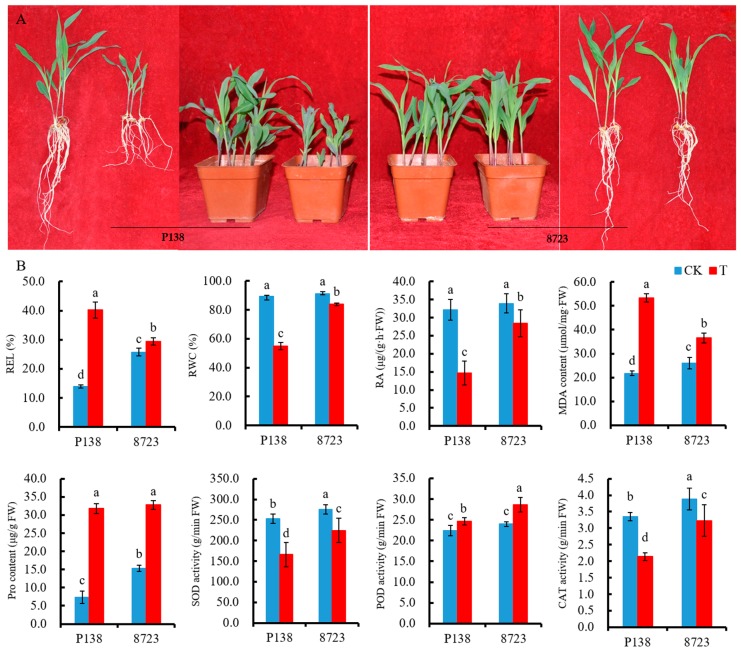
Seedling plant growth and physiological indexes (**A**) Seedling plant growth of P138 and 8723 under control conditions and 180 mM NaCl stress; (**B**) Seedling physiological indexes of P138 and 8723 under control conditions and 180 mM NaCl stress. Data are presented as the mean ± standard deviation. Lowercase letters indicate a significant difference at the 5% level. Abbreviations: CK: under control conditions, T: under 180 mM NaCl stress, REL: relative electrolyte leakage, RWC: relative water content, RA: root activity, MDA: malondialdehyde, Pro: proline, SOD: superoxide dismutase, POD: peroxidase, CAT: catalase.

Seedling morphological characteristics and physiological responses of P138 and 8723 were used to study salt stress. Plants of both varieties exhibited better growth status under control conditions, and a clear difference could be observed between two varieties under salt stress. After salt stress, 8723 grew more robustly than P138 and there was no obvious change from the control, while P138 decreased plant height and the roots were severely shortened (Figure 1A). Relative electrolyte leakage (REL), relative water content (RWC), root activity (RA), malondialdehyde (MDA) content, proline (Pro) content, superoxide dismutase (SOD) activity, peroxidase (POD) activity and catalase (CAT) activity were used as indexes to evaluate the salt tolerance of two varieties under salt stress. Compared with the control, the REL, MDA content, Pro content, and POD activity of the two varieties increased under salt stress. The REL and MDA content of 8723 was lower than that of P138, while Pro content and the POD activity of 8723 was higher than that of P138. Under salt stress, the RWC, RA, and SOD activity and CAT activity of the two varieties both decreased, while the RWC, RA, SOD activity and CAT activity of 8723 was higher than that of P138 (Figure 1B). In summary, these indicators in P138 were more strongly affected by salt stress.

### 2.2. iTRAQ-Based Protein Identification at the Maize Seedling Stage

**Figure 2:**
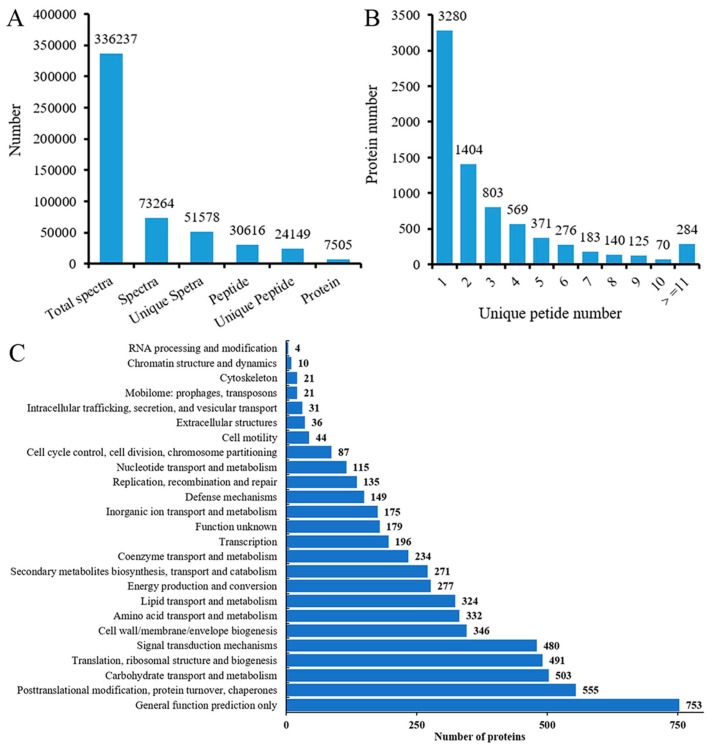
Statistics of root protein identification results: (**A**) Protein identification overview (**B**) Distribution of unique peptide number. False discovery rate (FDR) ≤ 1%. (**C**) COG annotation analysis of all proteins.

Quantitative proteomic analysis of seedling roots from salt-sensitive P138 and salt-tolerant 8723 were performed using the iTRAQ method. We obtained a total of 336237 spectra. 30616 peptides and a total of 7505 proteins were identified with 1% false discovery rate (FDR) (Figure 2A, Table S1). The proteins made up of more than 11 peptides accounted for only 3.7% of the total protein number, and the rest of the proteins were less than 10 peptides long, and the number of proteins decreased with the increase of the number of matching peptides (Figure 2B). In addition, Cluster of Orthologous Groups of Proteins (COG) analysis divided these proteins into 25 categories (Figure 2C). Most of the DEPs were clustered in the “general functional prediction only” category (13.1%), followed by posttranslational modification, protein turnover, and chaperones (9.6%).

### 2.3. Comparison of Differentiallly Expressed Genes in P138 and 8723 under NaCl Stress

**Figure 3:**
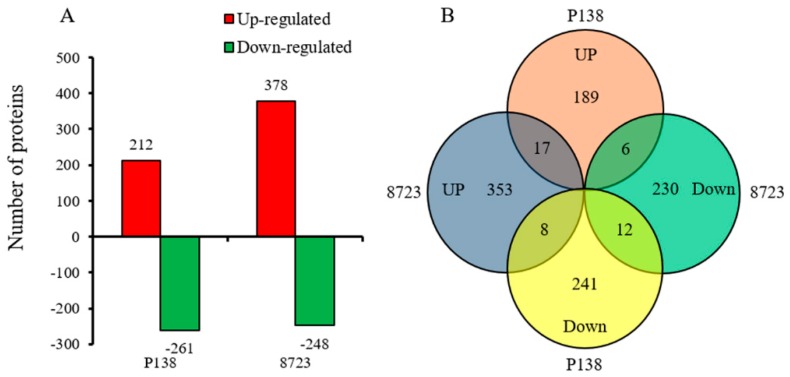
Comparative analysis of differentially expressed proteins in two maize varieties. (**A**) Number of up-regulated and down-regulated differentially expressed proteins (DEPs). (**B**) Venn diagram of root DEPs in two maize varieties.

From the iTRAQ identified proteins in two maize varieties, based on fold change > 1.2 and *p*-value < 0.05, a total of 1056 significant DEPs were identified (Figure 3A). Compared to the control, 473 DEPs were identified in P138 under salt stress, of which 212 were up-regulated and 261 were down-regulated (Tables S2 and S3), while 626 DEPs were identified in 8723, of which 378 were up-regulated and 248 were down-regulated (Tables S4 and S5). In Venn diagram analysis of root DEPs in two maize inbred lines under salt stress, 17 DEPs were commonly up-regulated in P138 and 8723, and 12 DEPs were commonly down-regulated. While 8 DEPs were up-regulated in 8723 but down-regulated in P138, 6 proteins were down-regulated in 8723 but up-regulated in P138 (Figure 3B). There were 43 differentially expressed proteins identified in two maize seedling roots under salt stress (Tables S6 and S7).

### 2.4. GO and KEGG Enrichment of the DEPs

**Figure 4:**
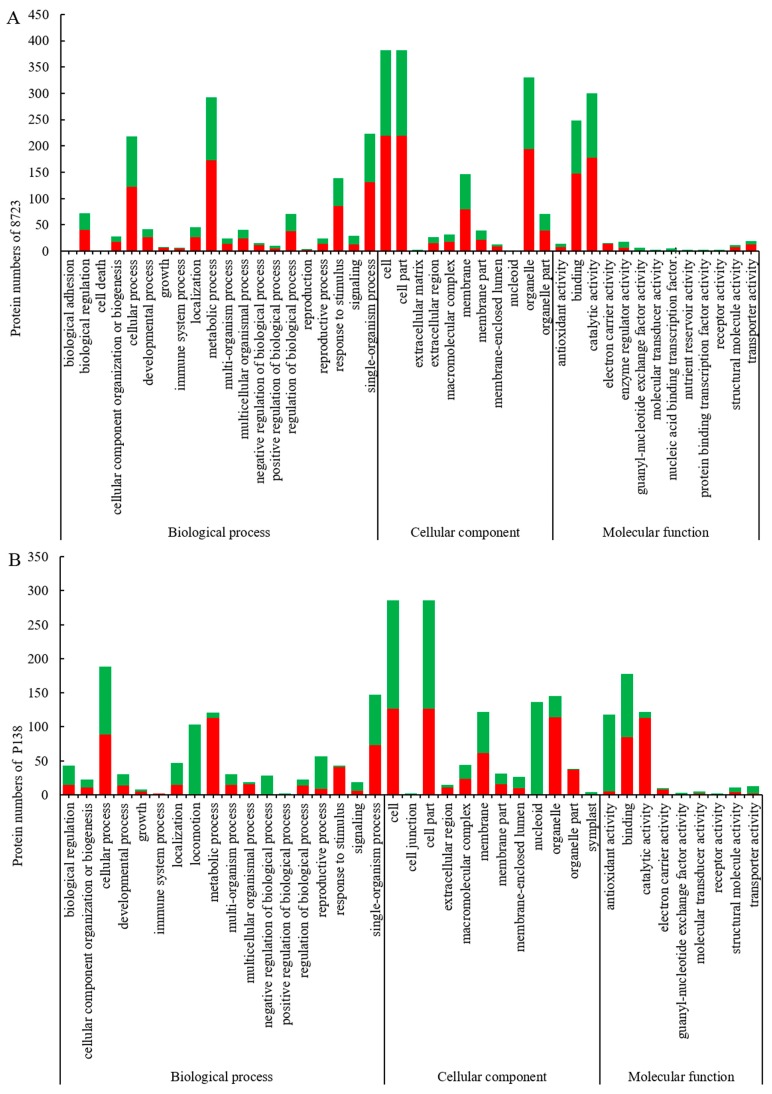
(**A**) Gene ontology (GO) annotation of DEPs in 8723. (**B**) GO annotation of DEPs in P138. Red bars represent increased protein abundances and green represent decreased.

To deduce the functionality and biological processes associated with the identified DEPs in the maize genotypes, gene ontology (GO) analysis and Kyoto Encyclopedia of Genes and Genomes (KEGG) enrichment were performed. GO analysis of identified DEPs can be divided into three categories: Cellular component, Molecular function, and Biological processes (Figure 4). A GO functional analysis showed that there were 36 GO terms such as response to stimulation, metabolic process, organelle and antioxidant activity sharing between 8723 and P138. For almost all of these common GO terms, the increased and decreased DRPs in 8723 were more than P138. There were eight unique terms in 8723, such as biological adhesion, cell death, reproduction, extracellular matrix, enzyme regulator activity, protein binding transcription factor activity and so on, whereas GO terms related to locomotion, cell junction and symplast were specifically enriched in P138 (Figure 4A,B).

In addition, compared to the control, pathway enrichment analysis of DEPs in the two varieties following salt treatment were summarized in Table 1. The 79.07% DEPs in 8723 were distributed in 110 pathways, eight of which were significantly enriched (*p* < 0.05), mainly concentrated in phenylpropanoid biosynthesis (5.25%) and starch and sucrose metabolism (5.05%). However, the 79.92% DEPs in P138 were distributed in 105 pathways. Intriguingly, only the nitrogen metabolism pathway was significantly enriched (*p* < 0.05).

### 2.5. Protein-Protein Interactions

**Figure 5:**
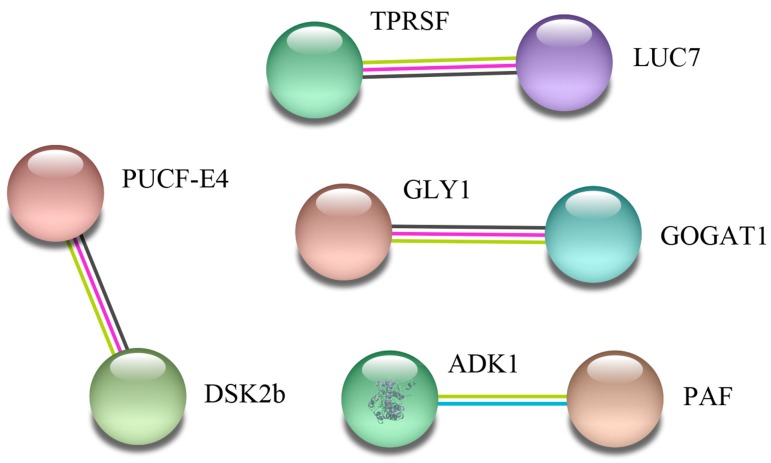
A protein interaction network of differentially regulated proteins between 8723 and P138 seedlings under salt stress. The network was built using a String program (http://www.string-db.org/), with confidence greater than 0.5. The nodes represent the protein, and the line color indicates the type of interaction evidence.

To determine how maize root cells transmit salt stress signals, further analysis of 43 differentially expressed proteins in the two varieties was performed using the String 11.0 database with a confidence score higher than 0.5. Four groups of proteins interacting with each other were identified in the two maize inbred lines (Figure 5). The first group includes: Tetratricopeptide repeat (TPR)-like superfamily protein (TPRSF) and LUC7 related protein (LUC7). These proteins are associated with secondary metabolite biosynthesis. The second group includes: ubiquitin domain-containing protein DSK2b (DSK2b) and putative ubiquitin conjugation factor E4 (PUCF-E4). These proteins are involved in ubiquitin-dependent proteolysis pathways. In the third protein network group, Glutamate synthase 1 (GOGAT1) interacted with Serine hydroxymethyltransferase (GLY1). As expected, they were related to amino acid biosynthesis. The fourth group includes: adenylate kinase (ADK1) and a putative apyrase family protein (PAF). These proteins are involved in regulating energy level.

### 2.6. qRT-PCR Verification

**Figure 6:**
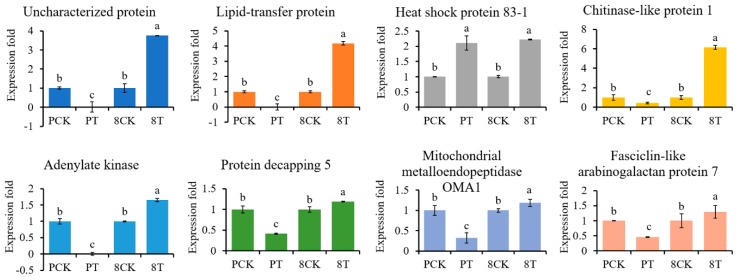
qRT-PCR analysis of gene expression fold change for eight proteins. PCK: P138 under normal conditions, PT: P138 under 180 mM NaCl stress, 8CK: 8723 under normal conditions, 8T: 8723 under 180 mM NaCl stress. Data are presented as the mean ± standard deviation. Lowercase letters indicate a significant difference at the 5% level.

To evaluate the reliability of the results of protein sequencing, we used qRT-PCR to analyze expression for eight genes, which were up-regulated in 8723 but down-regulated in P138 under salt stress (Figure 6). The transcript levels of seven genes were in agreement with the iTRAQ results, accounting for 87.5% of the analyzed genes, including lipid transfer protein and adenylate kinase 1. Only heat shock protein 83-1 showed the opposite trend from the iTRAQ results. This indicates that the results of our study reliably identify expression changes.

## 3. Discussion

### 3.1. Physiological Responses in Two Maize Inbred Lines under Salt Stress

Salt stress can seriously inhibit the growth and development of plants [20]. In this study, we found that the growth of maize seedlings was significantly hindered following salt stress. Further, the inhibition level of salt-sensitive P138 was more obvious, compared to the relatively salt-tolerant line 8723. The results showed that the inhibition degree of salt stress on maize growth was related to the characteristics of the maize plant itself.

The REL reflects the degree of damage to the plant cell membrane under osmotic stress. When a plant is subjected to salt stress, a lower REL indicates that the plant is more resistant to salt stress [21]. We found that 8723 has a lower REL than P138 under NaCl stress, which indicated that the cell membrane of 8723 suffered less damage under salt stress and had a strong salt tolerance. High relative water content (RWC) can help plants resist physiological drought caused by salt stress [14]. We found that the root system in salt-tolerant 8723 maintained a higher RWC than salt-sensitive P138 under salt stress, which suggested that 8723 protects against the physiological drought caused by salt stress through maintaining a high RWC. High root activity (RA) is an important indicator of resistance to stress, and RA will increase under abiotic stress [8]. In this study, 8723 maintains higher RA compared to P138 under salt stress, which indicates that the high RA of 8723 was one of the reasons for resistance to salt stress. Reactive oxygen species (ROS) affect the function of many cells by destroying nucleic acids, oxidizing proteins, and causing lipid peroxidation [22]. The antioxidant system of superoxide dismutase (SOD), catalase (CAT), and other enzymes can neutralize the accumulation of ROS induced by stress [23]. POD can maintain balance by removing excessive ROS and enhance plant tolerance to stress [24]. MDA is the product of lipid metabolism, and its content can directly reflect the degree of membrane damage caused by stress [25]. Proline, as a powerful antioxidant, can protect plants from reactive oxygen species caused by abiotic stress [26]. In our study, we found that the MDA content and Pro content of 8723 was lower than that of P138, but the SOD activity, CAT activity, and POD activity of 8723 was higher than that of P138 under salt stress. These results showed that salt-tolerant 8723 could maintain lower REL, MDA content, Pro content and higher RWC, RA, SOD activity, CAT activity, and POD activity to resist the damage caused by salt stress and enhance salt resistance.

### 3.2. Common Changes to the Metabolic Mechanism of 8723 and P138 under Salt Stress

Venn diagram analysis showed that 29 differentially expressed proteins showed the same trend under salt stress (Table S6) across both maize lines, which reflected the commonality of metabolic changes in resistance to salt stress.

#### 3.2.1. Up-regulation of Lipid-Metabolism Related Proteins Could Contribute to Increased Signaling

Lipids are an important membrane component and are involved in many metabolic pathways, such as energy storage and membrane synthesis [27]. Lipid phosphate phosphatases (LPPs) are complete membrane proteins that can significantly alter the signaling balance between the phosphate esters and their dephosphorylated products through glycerolipids and sphingolipids [28]. Previous studies have found that lipid metabolism plays an important role in the aboveground organs and root tissues of salt-tolerant barley (*Hordeum vulgaries* L.) [29]. In this study, we found that one LPP3 (tr|A0A3L6DLG4) was up-regulated in two maize inbred lines under salt stress, which indicated that LPP3 may respond positively to salt stress through increased signaling in maize.

#### 3.2.2. Reducing Ammonia/Free Branched Chain Amino Acid Toxicity to Enhance Maize Salt Tolerance

Protein synthesis is indispensable for salt stress adaptation [30]. Under salt stress, the up-regulation of glutamate synthase in the nitrogen metabolism pathway can increase the synthesis of glutamate and reduce the excessive accumulation of ammonia, which is beneficial for plants to respond to salt stress [31]. Here, we found that a glutamate synthase 1 (GOGAT1) (tr|A0A1D6MBZ9) was upregulated in two maize inbred lines under salt stress, indicating that the increased accumulation of this protein is beneficial to maize resistance to salt stress. Branched chain amino acids are synthesized from branched chain ketoacids in plants [32]. Branched-chain amino acid aminotransferase (BCAT) may be involved in the degradation of branched-chain amino acids and maintain the low and non-toxic levels of free branched chain amino acids under abiotic stress to resist stress injury [33]. Here, we also identified BCAT (tr|A0A3L6DDE5) as upregulated under salt stress. This shows that BCAT decomposes free amino acids in maize plants under salt stress to alleviate the injury caused by salt stress.

#### 3.2.3. Up-Regulation of Stress Defense Related Proteins to Improve Salt Tolerance

There may be cross-tolerance in plants; for example, a specific protein is often upregulated in response to one stressor, and this same protein may also provide resistance to other stresses. Receptor-like protein kinase (RLKs) are the largest gene family in plants, and many RLKs are involved in the response to abiotic stresses including salt, drought, and cold stress [34]. In this study, we identified one RLK (tr|B6TKJ6) which was up-regulated in two maize inbred lines under salt stress. Therefore, we speculate that RLKs enhance the salt tolerance of maize. HVA22-like protein is a dehydrated protective protein that is resistant to drought stress [35]. In this study, HVA22-like protein a (HLPa) (tr|A0A3L6EB16) was up-regulated under salt stress, so we speculate that HLPa may play a positive role both in salt stress and in drought stress.

#### 3.2.4. Redox Related Proteins

Salt stress disrupts ion stability in plants, leads to osmotic imbalance, and further triggers oxidative damage, which is harmful to plant growth and development [36]. Proteomic analysis of 8723 and P138 showed that redox reactions were involved in maize response to salt stress. Cytochrome P450 is an important mono-oxygenase involved in xenobiotic metabolism and steroid biosynthesis [37]. Previous studies have found that cytochrome P450 was up-regulated in *Arabidopsis thaliana* under salt stress [38]. We found that cytochrome P450 11 (P45011) (tr|A0A1D6NI59) was also up-regulated in two maize inbred lines under salt stress, which may indicate that P450 11 is a salt stress active protein. Feredoxin NADP-dependent oxidoreductase (NADP-DOR) is a flavin adenine dinucleotide-binding enzyme, which is encoded by the small nuclear gene family and commonly found in higher plants [39]. In *Populus simonii*, NADP-DOR was up-regulated under abiotic stress [40]. Here, a NADP-DOR (tr|B6TDE0) was up-regulated under salt stress. This indicates that NADP-DOR is a salt stress up-regulation protein. In order to protect cells from oxidative damage caused by excessive reactive oxygen species (ROS), plants usually adopt a series of complex defense mechanisms, such as enhancing the activity of antioxidant enzymes [41]. Peroxidase (POD) enzyme is involved in ROS signaling and redox reactions and high POD content can increase the salt tolerance of plants [8]. The accumulation of this enzyme (tr|B4G0X5) is consistent with the results of physiological observations. This indicated that maize enhanced salt tolerance by increasing POD content. In addition, many proteomic studies have confirmed that redox related proteins are involved in salt stress in plants, including rice [42], maize [43], barley [44] and Sugar Beet (*Beta vulgaris* L.) [45]. In general, oxidation and reduction reactions may improve the salt tolerance of maize.

#### 3.2.5. Energy-Related Proteins Respond to Salt Stress

Plants need to regulate different physiological and metabolic processes to reduce the harm of salt stress, and a constant energy supply is essential [46]. Granule bound starch Synthase 1 (GBSS1) is regulated at the transcriptional and post-transcriptional levels, which is responsible for the synthesis of amylose in cereals [47]. Previous studies have found that 200 mM NaCl stress decreased the expression of GBSSI and GBSSII genes in rice [48]. However, Granule-bound starch synthase 1 (GBSS1) was up-regulated in two maize inbred lines under 180 mM stress. This may be related to different levels of salt treatment or different materials. Glycosyltransferase is an enzyme that catalyzes the transfer of sugar chains from activated donor molecules to specific recipient molecules [49]. The accumulation of glycosyltransferase is beneficial to the response of plants to salt stress [50]. In this study, we found that one glycosyltransferase (tr|A0A1D6EWJ3) was up-regulated under salt stress. This shows that the accumulation of the enzyme plays a positive role under salt stress. In addition, we found a putative apyrase family protein (APY) was up-regulated in two maize inbred lines under salt stress. GO analysis showed that APY could catalyze the hydrolysis of triphosphate and diphosphate nucleosides. Previous studies have found that plant apyrase plays an important role in plant abiotic and abiotic stresses [51]. Therefore, we suggested that APY may improve the salt tolerance of maize by increasing its energy supply.

#### 3.2.6. Signal Transduction Related Protein

The response of plants to abiotic stress is to consolidate their growth and development by regulating complex signal networks for adaptation to stress [52]. WRKY71 is rapidly induced by H_2_O_2_, ABA, and mannitol, which are involved in the regulation of abiotic stress responses in plants [53]. In this study, the WRKY transcription factor WRKY71 (tr|A0A3L6EHD4) was down-regulated under salt stress. We suggest that WRKY71 may negatively regulate the response to salt stress. Ent-isokaurene C2-hydroxylase (Ent-IKC2) may be related to the biosynthesis of gas and indoleacetic acid [54]. Further, previous studies have found that exogenous indoleacetic acid can effectively alleviate the adverse effects of salt stress on maize plants [55]. In this study, we found one Ent-IKC2 (tr|A0A3L6EVD8), which was up-regulated in two maize inbred lines under salt stress. These results suggested that Ent-IKC2 may enhance the biosynthesis of indoleacetic acid to repair the damage caused by salt stress. Highly conserved nuclear pore complexes mainly regulate the exchange of macromolecules including RNA and proteins, which play an important role in the regulation of abiotic stress in plants [56]. Here, we observed that a nuclear pore complex protein (NPCP) (tr|A0A1D6PPU7) was up-regulated in two inbred lines, indicating that the protein positively responded to salt stress. G subfamily ABC transporters play a role through Na/K balance and actively respond to salt stress in rice [57]. Here, an ABC transporter G family member 34 (tr|A0A1D6LCS6) was up-regulated under salt stress. This suggested that the protein can alleviate the osmotic stress by regulating ion balance. CCR4-associated factor 17 (CCR4F17) is an evolutionarily conserved protein complex, which is involved in the regulation of transcription and gene degradation and plays an important role in plant growth and defense [58]. There is one probable CCR4F17 (tr|A0A3L6E1N9) which was down regulated under salt stress. We speculate that CCR4F17 may negatively regulate the response to salt stress. In addition, we found that a ras related protein, RABH1b (tr|B4G1R3), was down-regulated in two maize inbred lines under salt stress and involved in the signal transduction mediated by small molecule GTPase. Therefore, we speculate that this protein may be a salt stress negative regulatory protein. These results suggest that plants respond to salt stress by regulating the protein pool through complex signal networks.

#### 3.2.7. Down-Regulation of Ubiquitin-Associated Proteins’ Response to Salt Stress

The ubiquitin-dependent proteolysis pathway can degrade most proteins and is the main mechanism of protein degradation in eukaryotic cells [59]. Ubiquitin is involved in the post-translational modification of proteins in eukaryotes and plays an important role in plant environmental stress [60]. In this study, we found that three ubiquitin-related proteins were down-regulated under salt stress, including ubiquitin domain-containing protein DSK2b (DSK2b) (tr|A0A1D6K7R4), putative ubiquitin conjugation factor E4 (UBFE4) (tr|A0A1D6K601), and putative E3 ubiquitin-protein ligase LUL3 (LUL3) (tr|A0A3L6EHX5). UBFE4 participates in ubiquitin-dependent protein catabolism to protect itself from unnecessary protein degradation under salt stress. The down-regulation of E3 ubiquitin ligase PUB30 may increase salt tolerance by regulating the degradation of BRI1 kinase inhibitor 1 and brassinosteroid signaling in *A. thaliana* [61]. Therefore, we believe that the down-regulation of ubiquitin-related proteins may reduce the degradation of essential proteins in maize seedlings under salt stress, thus improving the salt tolerance of maize seedlings.

### 3.3. Differences in Molecular Mechanisms of Response to Salt Stress between Two Inbred Lines

Venn diagram analysis showed that 14 differentially expressed proteins exhibited a different direction of accumulation under salt stress (Table S7), and these proteins reflected the difference of salt tolerance between two maize inbred lines.

#### 3.3.1. Increasing Lipid Transport to Enhance 8723 Salt Tolerance

Lipid transfer protein (LTP) is involved in lipid metabolism, and it is up-regulated to improve the salt tolerance of tomato (*Solanum lycopersicum*) [62]. There is one LTP (tr|A0A1D6GTY3) up-regulated in 8723 but down-regulated under salt stress, which indicated that LTP played an important role in the recovery of maize under salt stress.

#### 3.3.2. Stronger Defense against Salt Stress in 8723

Heat shock proteins play an important role in protecting plants from stress by preserving other proteins in their functional conformations [63]. Previous studies have found that heat shock protein had heat, salt, and drought resistance in transgenic *A. thaliana* [64]. We found that Heat shock protein 81-3 (HSP81-3) (tr|A0A3L6FZE4) was up-regulated in salt-tolerant 8723 under salt stress. We suggested that the increased accumulation (up-regulation) of HSP81-3 could be regarded as a crucial defensive response of 8723 against salt stress. Fasciclin-like arabinogalactan proteins (FLAs) are a subclass of arabinogalactan proteins [65], and Zang et al. [66] believed it was usually involved in abiotic stress responses in higher plants. In this study, FLA7 (tr|A0A1D6ES24) was up-regulated in salt-tolerant 8723 under salt stress. Therefore, we believe that the accumulation of FLA7 is beneficial to the salt tolerance of plants.

#### 3.3.3. Enhanced Nucleic Acid Biosynthesis to Resist Salt Stress

Adenylate kinase (AKS) balances adenylate by transferring a phosphate group from ATP to AMP to catalyze the formation of ADP [67]. In addition, previous studies showed that NaCl could significantly promote the activity of AKS in salt-sensitive rice (*Oryza sativa* L.) roots, but had no effect on salt tolerance [68]. However, in this study, AKS (tr|B6STL7) was up-regulated in salt-tolerant varieties under salt stress. We speculate that AKS may enhance the salt tolerance of maize by increasing energy levels. Protein decapping 5 (DCP5) is necessary for translation inhibition and P-body formation, and plays an indirect role in mRNA degradation [69]. We found that a DCP5 (tr|A0A1D6LXV8) was up-regulated in 8723 but down-regulated under salt stress. This suggested that DCP5 may respond to salt stress through the degradation of mRNA.

#### 3.3.4. Down-Regulation of ROP Guanine Nucleotide Exchange Factor Responses to Salt Stress

Guanine nucleotide exchange factor is an activator of Rop (Rho of Plants) GTP enzyme, which can promote the exchange between GDP and GTP [70]. We found that a Rop guanine nucleotide exchange factor 1 (RGNEF1) (tr|A0A1D6IB98) was down-regulated in 8723 but up-regulated in P138 under salt stress. This indicated that RGNEF1 down-regulated the salt stress response.

#### 3.3.5. Enhancing Salt Tolerance by Reducing Cell Membrane Damage

Previous studies have found Serine hydroxymethyltransferase 1 (SHT1) to be mainly involved in photorespiratory pathways, and it also plays an important role in controlling cell damage induced by abiotic stresses such as high light and salt [71]. One SHT (tr|B6T7J7) was up-regulated in salt-tolerant 8723 but down-regulated in P138, which suggested that SHT may be a salt-responsive negative regulatory protein. The vascular bundle endodermis and ectodermal casparian strip in plant roots play an important role in preventing salt from entering the middle column non-selectively along the apoplast [72]. Casparian strip protein (CASP) was reported to play an active role in salt stress in maize [73]. However, we found that CASP-like protein (tr|K7UJ00) was down-regulated in salt-tolerant maize and belongs to the casparian strip membrane proteins family. It can be speculated that CASP-like protein may be negatively regulated under salt stress in the casparian strip.

### 3.4. Analysis of Metabolic Pathway in P138 and 8723 under Salt Stress

#### 3.4.1. MAPK Signaling Pathway in 8723 Response to Salt Stress

**Figure 7:**
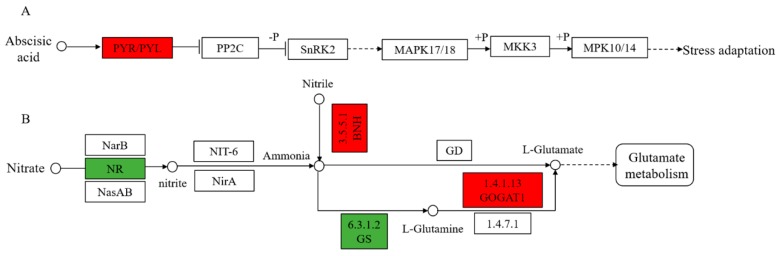
(**A**) MAPK signaling pathway in the response of 8723 to salt stress. (**B**) Nitrogen metabolic response to salt in P138. The red box indicated that the protein was up-regulated under salt stress, and green boxes represent down-regulated proteins under salt stress. Abbreviation: PYR/PYL: pathogenesis-related protein 10, NR: nitrate reductase, GS: glutamine synthetase, GOGAT1: Glutamate synthase 1, NBH: Bifunctional nitrilase/nitrile hydratase NIT4.

The MAPK cascade (signal transduction mechanism) plays a very important role in intracellular pathogen immunity and abiotic stress signal transduction. The MAPK cascade plays a key role in enzyme activation and inactivation through phosphorylation/dephosphorylation, which allows rapid and specific signal transduction and amplification of external stimuli [74]. In the abscisic acid (ABA) pathway of the MAPK cascade, we identified a pathogenesis-related protein 10 (PAP10) which was up-regulated in 8723 but constant in P138, which is involved in the synthesis of abscisic acid (Figure 7A). In addition, we also found that a chitinase-like protein 1 (CHP1) is involved in coercive defense in MAPK. As an important signaling molecule, salt stress or water deficit can induce the rapid accumulation of ABA in plant tissues and improve the ability of plants to resist salt stress. Chitinase-like protein (CLP) can prevent the excessive accumulation of Na ions, thus promoting the salt tolerance of *A. thaliana* [75]. Therefore, up-regulation of PAP10 and CHP1 means that 8723 improves salt tolerance by accumulating ABA and preventing excessive accumulation of Na ions.

#### 3.4.2. Nitrogen Metabolic Response to Salt Stress

The metabolic response mechanism of plants to abiotic stress is a complex process [76]. Many studies have found that nitrogen metabolism plays an important role in the plant response to salt stress [77,78,79]. In our study, only the nitrogen metabolism pathway was identified in salt-sensitive P138 (Figure 7B). Previous studies have found that the nitrate reductase (NR) activity increases under salt stress in maize [80]. In the reaction of nitrate reduction to nitrite, we identified that five cytochrome b5 genes were down-regulated under salt stress, which may affect nitrate reduction. During the reaction of nitrile to ammonia, a bifunctional nitrilase/nitrile hydratase NIT4 (NBH) is up-regulated in P138 but constant in 8723 under salt stress. In the pathway of l-glutamine ammonia synthesis, it was found that a glutamine synthetase (GS) was down-regulated, this leads to excessive accumulation of ammonium. In the pathway of producing l-glutamate, a Glutamate synthase 1 (GOGAT1) was up-regulated. The transformation from inorganic nitrogen to organic nitrogen is a protective strategy for detoxifying ammonia in plants [81]. Excessive accumulation of ammonia in plant cells can produce toxic effects [82]. Therefore, we speculate that the main reason for the poor salt tolerance of P138 is that the transformation from inorganic nitrogen to organic nitrogen is destroyed by salt stress and the formation of ammonia toxicity.

### 3.5. Proposed Molecular Model of 8723 Salt Tolerance

**Figure 8:**
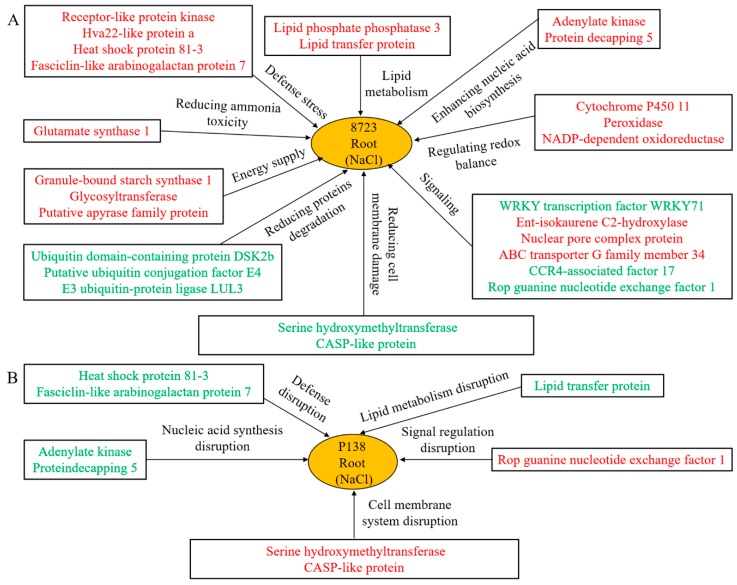
Molecular models of salt tolerance in maize seedling roots of: (**A**) tolerant inbred line 8723 and (**B**) sensitive line P138. The red font represents the up-regulated proteins, and the green represents the down-regulated proteins.

Based on the comparative analysis of proteomics and physiological differences between two inbred lines with varying tolerance under salt stress, we developed the following salt tolerance model of maize (Figure 8). The two maize inbred lines showed a common mechanism under salt stress: up-regulation of lipid metabolism-related proteins to increase signal transduction and resist the injury of salt stress. Relieving the toxicity of ammonia/overaccumulation of free branched chain amino acids, such as GOGAT1, BCAT expression was up-regulated in response to salt stress. Up-regulation of the stress response proteins RLK and HVA22 enhanced plant cross tolerance. Redox is the main mechanism of the plant response to abiotic stress, including the up-regulation of POD, NADP-DOR, cytochrome P450 11, scavenging of ROS, stabilizing the ion balance in plants, and alleviating the damage of salt stress to plants. Maintaining an energy supply is indispensable for plants to reduce salt stress injury, including mechanisms such as GBSS1, glycosyltransferase, APY accumulation, and regulating energy balance in plants. Down-regulation of Ubiquitin-associated proteins can reduce the degradation of unnecessary proteins. In addition, 8723 is more salt-tolerant due to the up-regulation of lipid transporters and increased lipid metabolism. Stress defense-related proteins, such as heat shock proteins and FLAs were up-regulated, increasing 8723 salt tolerance. Finally, 8723 can improve salt tolerance by increasing nucleic acid biosynthesis and signal transduction levels. 8723 can also better regulate osmotic pressure balance and reduce the damage of salt stress to the membrane.

## 4. Materials and Methods

Salt-tolerant 8723 and salt-sensitive P138 were used to study the mechanism of salt tolerance in maize. In addition, 8723 pedigree derived from the cross between U8112 and Ye107, and P138 was derived from the progeny material of the American hybrid P78599. When selecting these two inbred lines, we screened the seedling physiological indexes of 13 maize inbred lines under salt stress. This finding was supported by previous experiments [83,84]. Seeds were surface-sterilized with 0.5% NaClO for 10 min, and washed five times with distilled water, then soaked for 12 h in either deionized water (as control, CK) or 180 mM NaCl solution (T), and sown in pots (15 cm × 13 cm, 10 seedlings per pot) with three biological replicates per treatment. After sowing, seeds were cultured in the greenhouse, at a temperature of 25 ± 2 °C, 12 h light, 600 µmol/s^−1^·m^−2^ light intensity, 60% relative humidity, and 50 mL deionized water was added every two days. Further, 50 mL 180 mM NaCl solution was added every three days, beginning when the control leaves grew to the three-leaf stage (about ten days). For proteomic analysis, roots of each treatment were washed with distilled water and then quickly placed in liquid nitrogen. The sample was stored at −80 °C for further use. Two independent biological replicates were sampled for proteomic analysis.

### 4.1. Measurement of Physiological Parameters

The physiological indicators of the roots were determined at the three-leaf stage. The relative water content (RWC) was measured as described by Galmés et al. [85], the relative electrolyte leakage (REL) was estimated according to Liu et al. [86], root activity (RA) was measured as described by Baozhang et al. [87], the proline (Pro) content was quantified according to a ninhydrin-based colorimetric assay [88], malondialdehyde (MDA) content was measured using the thiobarbituric acid assay method [89], and superoxide dismutase (SOD) activity was determined by the nitroblue tetrazolium photoreduction [90]. Catalase (CAT) activity was determined by the H_2_O_2_ mothed [91], and peroxidase (POD) activity was estimated by the guaiacol method [92].

### 4.2. Protein Analysis

#### 4.2.1. Protein Extraction and iTRAQ Labeling

iTRAQ analysis was implemented at BGI (Shenzhen, China). Total protein was extracted from seedling roots under the treatment of CK and T according to the company’s method [93,94]. The protein concentration was determined using the Bradford dye-binding assay [95]. For each sample, the solution containing 100 µg of protein was transferred to a new tube. Trypsin Gold (Promega, Madison, WI, USA) with the ratio of protein:trypsin = 40:1 was added and hydrolyzed at 37 °C for 4 h. Then, Trypsin Gold was added once again with a ratio of protein:trypsin = 40:1, and digested for 8 h at 37 °C. After trypsin digestion, desalination was carried out with the StrataXC18 column (Phenomenex, Torrance, CA, USA), and vacuum drying was carried out according to the manufacturer’s protocol. These peptides are dissolved in 0.5M TEAB by vortex. When the iTRAQ labeling reagent reaches room temperature, it is transferred and combined with the appropriate sample. The peptides from 8723 and P138 were labeled according to the manufacturer’s protocol for 8-plex iTRAQ reagent (Applied Biosystems, Foster city, CA, USA). The iTRAQ tag is shown in Table S8.

#### 4.2.2. Strong Cation Exchange (SCX) Fractionation LC-ESI/MS Analysis Based on Triple TOF 5600

A LC-20AB higher performance liquid chromatography pump system (Shimadzu Kyoto, Japan) with a high pH RP column was used to separate SCX chromatography. The iTRAQ labeled polypeptide mixture was recomposed with 4ml buffer A (5% ACN, pH 9.8) and loaded into a Ultremex SCX column containing 5 µm particles (4.6 × 250 mm). At the flow rate of 1 mL/min, A buffer gradient eluted for 10 min, 5–35% buffer B (95% ACN, pH 9.8) for 40 min, 35–95% buffer B for 1 min. The system was then maintained at 100% buffer B for 1 min before equilibrating with buffer A for 10 min before the next injection. The eluting degree was monitored by the absorbance at 214 nm, and the components were collected every other minute. Combined with a chromatographic elution peak diagram, the eluted peptides were divided into 20 components, desalted by the Strata X C18 column, and dried in vacuum.

#### 4.2.3. LC-ESI/MS Analysis Based on Triple TOF 5600

Each component was resuscitated in A buffer (2% ACN and 0.1% FA) and centrifuged at 20,000 *g* for 10 min. Using a LC-20AD nanometer high performance liquid chromatograph (Shimadzu, Japan), 10 µl of the supernatant was fixed on a C18 column with a flow rate of 5 µL/min and continuously for 8 min. The internal analysis C18 column (inner diameter 75 µm, column size 3.6 µm, column length 15 cm) was used for separation. The gradient was separated at the flow rate of 300 nL/min. 5% buffer B ran for 8 min, 8% to 35% B buffer (98% ACN and 0.1% FA) for 35 min, 5 min linear gradient to 60%, 2 min linear gradient to 80% and maintained for 4 min, and finally returned to 5% in 1 min.

The data are collected using the TripleTOF5600 system (SCIEX, Framingham, Massachusetts, USA) equipped with a nanometer III source (SCIEX, Framingham, MA, USA), a quartz needle tip as the transmitter (New Objectives, Woburn, MA), and controlled by software analyst 1.6 (AB SCIEX, Concord, ON) During data acquisition, the parameters of the mass spectrometer are as follows: ion spray voltage is 2300V, the nitrogen pressure is 30psi, the atomization gas is 15, and the temperature at the spray interface is 150 °C. High sensitivity mode is used for the whole data acquisition. The cumulative time of MS1 is 250 Ms and the scanning quality range is 350~1500Da. According to the intensity measured by MS1, up to 30 product ion scans are collected if the product ion scan exceeds the threshold of 120 counts per second and the charge state is 2 + to 5 +. The Q2 transmission window of 100 Da is 100%. Through the detection of four anodes/channels of 40 GHz multi-channel thermal conductivity detector, when the pulse frequency is 11 kHz, four-time bins are obtained for each scan. For iTRAQ data acquisition, collision can be adjusted to all precursor ions for collision-induced dissociation. Dynamic exclusion is set for 1/2 of the peak width (15s), and the precursor is refreshed from the exclusion list. The mass spectrometric data can be found by ProteomeXchange with identifier PXD014409.

#### 4.2.4. Database Search and Protein Quantification

First, the raw MS/MS data was converted to MGF format using the ProteoWizard tool msConvert. Then the protein was identified by Mascot search engine (Matrix Science, London, UK; version 2.3.02) and compared with the uniport (https://www.uniprot.org/, *Zea_mays*, 2017_03_24) database containing 140026 sequences. Mascot search parameters: MS/MS ion search is the search type, the mass values is monoisotopic, the fragment mass tolerance is 0.1 Da (ppm), the peptide mass tolerance of 0.05Da, only one uncut peptide is allowed in trypsin digestive juice, oxidized (M) and iTRAQ8plex (Y) were used as variable modifications, and carbamidomethyl (C), iTRAQ8plex (N-Term) and iTRAQ8plex (K) were used as fixed modifications. The screening reliability was more than 95% (*p* < 0.05) and FDR (False discovery rate) ≤1%. Each identified protein identification involved at least one unique polypeptide.

#### 4.2.5. RNA Extraction and qRT-PCR

Total RNAs were extracted from each seedling root of the treatment and control groups separately, according to the manufacturer’s protocol for the RNAsimple Total RNA Kit (Tiangen, Shanghai, China). Extracted RNA was reverse-transcribed into cDNA using FastKing cDNA (Tiangen, Shanghai, China). Specific PCR primers of the eight selected genes were designed using Primer5 (Table S2). Samples and standards were performed on a QuantStudio 5 Real-Time PCR System (Thermo Scientific, Massachusetts, USA) using the SuperReal PreMix Plus (SYBR Green) (Tiangen, Shanghai, China). Three biological replicates were included. Each Real-Time PCR was performed in a 20 µL reaction volume containing 10 µL SuperReal PreMix Plus, 6 µL ddH2O, 0.8 µL forward primer (10 µmol/L), 0.8 µL reverse primer (10 µmol/L), 0.4 µL ROX Reference Dye and 2 µL template cDNA. The PCR programs were run as follows: 10 min of pre-denaturation at 95 °C, 40 cycles of 15 s at 95 °C, 30 s at 60 °C, melt curve stage: 15 s at 95 °C, 1 min at 60 °C, and 15s at 95 °C. The transcript abundance of selected genes was calculated by 2^−ΔΔCt^ method and normalized with the results of the 18s gene [96]. The primers for RT-PCR are shown in Supplemental Table S9.

### 4.3. Bioinformatics Analysis

Each physiological parameter was examined with three biological replicates. All of the data were analyzed by SPSS 19.0, the results were expressed by mean ± standard deviation (SD), and Microsoft Excel 2016 was used to map.

For successfully identified proteins, according to the weighted average of peptide ionic strength labeled by iTRAQ, at least two unique spectra are needed to represent one protein. The relative expression of each protein was normalized by Mascot software. All the identified proteins were compared with the COG database (http://www.ncbi.nlm.nih.gov/COG/) by protein BLAST, and the corresponding COG annotation results were obtained. According to the basic functional and metabolic characteristics of Bevan et al. [97], all differentially expressed proteins were classified and GO annotation (http://geneontology.org/) was used to classify. The biological process, cellular composition and molecular function of differentially expressed proteins were enriched and analyzed. The metabolic pathways of differentially expressed proteins were enriched and analyzed by using KEGG pathway database. Proteins with fold-changes >1.2 or <0.83 (*p* < 0.05) were considered to be DEPs.

## 5. Conclusions

Salt stress tolerance is a complex phenomenon in plants, which occurs from the cellular level to the whole plant level. In this study, morphological, physiological, and proteomic analyses of salt-tolerant 8723 and salt-sensitive P138 were compared to elucidate the possible mechanisms of salt stress tolerance. Through proteomics analysis of iTRAQ technique, a total of 1056 DEPs were identified. Enrichment of 43 proteins changed significantly in the two varieties. In salt-stressed 8723, the DEPs were primarily associated with phenylpropanoid biosynthesis, starch and sucrose metabolism, and the MAPK signaling pathway. Intriguingly, the DEPs were only associated with the nitrogen metabolism pathway in P138. Compared to P138, the 8723 root response to salt stress could maintain stronger water retention capacity, synergistic effect of antioxidant enzymes, metabolism and energy supply capacity, osmotic regulation ability, signal transduction, ammonia detoxification ability, lipid metabolism and increased nucleic acid synthesis. This study provides new insights for further understanding the molecular mechanism of salt tolerance in maize.

## Figures and Tables

**Table 1:** Significant pathway enrichment analysis of differentially expressed proteins (DEPs) in 8723 and P138 (*p* < 0.05).

Maize Varieties and Experimental Treatment	Pathway	Number of DEPs	*p*-value	Pathway ID
8723 T vs. 8723 CK	Starch and sucrose metabolism	25 (5.05%)	0.005236	ko00500
	Glycerolipid metabolism	12 (2.42%)	0.005343	ko00561
	MAPK signaling pathway	16 (3.23%)	0.021084	ko04016
	Diterpenoid biosynthesis	4 (0.81%)	0.026879	ko00904
	Mannose type O-glycan biosynthesis	3 (0.61%)	0.039637	ko00515
	Phenylpropanoid biosynthesis	26 (5.25%)	0.044924	ko00940
	Cyanoamino acid metabolism	9 (1.82%)	0.046657	ko00460
	Galactose metabolism	11 (2.22%)	0.047682	ko00052
P138 T vs. P138 CK	Nitrogen metabolism	8 (2.12%)	0.000314	ko00910

## Supplementary Materials

Supplementary materials can be found at https://www.mdpi.com/1422-0067/20/19/4725/s1.

## Abbreviations

iTRAQ	Isobaric tags for relative and absolute quantitation
DEPs	Differentially expressed proteins
qRT-PCR	Quantitative real-time polymerase chain reaction
CK	Normal conditions
T	180 mM NaCl stress
REL	Relative electrolyte leakage
RWC	Relative water content
RA	Root activity
MDA	Malondialdehyde
Pro	Proline
SOD	Superoxide dismutase
POD	Peroxidase
CAT	Catalase
MAPK	Mitogen-activated protein kinase
FDR	False discovery rate
GO	Gene ontology
COG	Cluster of Orthologous Groups of proteins
KEGG	Kyoto Encyclopedia of Genes and Genomes
LPP3	Lipid phosphate phosphatase 3
NBH	Bifunctional nitrilase/nitrile hydratase NIT4
GS	Glutamine synthetase
NR	Nitrate reductase
PYR/PYL	Pathogenesis-related protein 10
AKS	Adenylate kinase
GOGAT1	Glutamate synthase 1
NADP-DOR	NADP-dependent oxidoreductase
ABA	Abscisic acid
ROS	Reactive Oxygen Species
LC-MS/MS	Liquid Chromatography-Electrospray Ionization-Tandem Mass Spectrometry
