# Supplementary material for: Comparative Proteomics of Salt-Tolerant and Salt-Sensitive Maize Inbred Lines to Reveal the Molecular Mechanism of Salt Tolerance
Source: Int J Mol Sci. 2019 Sep 24;20(19):4725. doi: 10.3390/ijms20194725 (PMC6801879; doi:10.3390/ijms20194725)
Supplement: Supplementary file 1 [file ijms-20-04725-s001.zip › ijms-546735-supplementary-final/Supplementary Legend.docx]

Supplementary Legend

Comparative Proteomics of Salt-tolerant and Salt-sensitive Maize Inbred Lines to Reveal the Molecular Mechanism of Salt Tolerance

Fenqi Chen ^1,†^, Peng Fang ^1,†^, Yunling Peng ^1,2,^*, Wenjing Zeng ^1^, Xiaoqiang Zhao ^2^,
Yongfu Ding ^1^, Zelong Zhuang ^1^, Qiaohong Gao ^1^ and Bin Ren ^1^

**Table S1.** Total maize seedling root proteins identified by iTRAQ.

**Table S2.** Proteins up-regulated in P138 seedling roots.

**Table S3.** Proteins down-regulated in P138 seedling roots.

**Table S4.** Proteins up-regulated in 8723 seedling roots.

**Table S5.** Proteins down-regulated in 8723 seedling roots.

**Table S6.** Differentially expressed proteins showed the same trend in salt-tolerant 8723 and salt-sensitive P138 under salt stress.

**Table S7.** Differentially expressed proteins showed the opposite trend in salt-tolerant 8723 and salt-sensitive P138 under salt stress.

**Table S8.** iTRAQ labeling information.

**Table S9.** Primer sequences used for qRT-PCR analysis in this article.
